# Culture-confirmed childhood tuberculosis in Cape Town, South Africa: a review of 596 cases

**DOI:** 10.1186/1471-2334-7-140

**Published:** 2007-11-29

**Authors:** H Simon Schaaf, Ben J Marais, Andrew Whitelaw, Anneke C Hesseling, Brian Eley, Gregory D Hussey, Peter R Donald

**Affiliations:** 1 Desmond Tutu Tuberculosis Centre, Department of Paediatrics and Child Health, Stellenbosch University, Cape Town, South Africa; 2 Tygerberg Children's Hospital, Cape Town, South Africa; 3 National Health Laboratory Service, Groote Schuur and Red Cross Children's Hospital, Cape Town, South Africa; 4 Department of Clinical Laboratory Sciences, University of Cape Town, Cape Town, South Africa; 5 Department of Infectious and Tropical Diseases, London School of Hygiene and Tropical Medicine, London, UK; 6 Red Cross Children's Hospital, Cape Town, South Africa; 7 School of Child and Adolescent Health, University of Cape Town, Cape Town, South Africa; 8 Institute of Infectious Diseases and Molecular Medicine, Faculty of Health Sciences, University of Cape Town, Cape Town, South Africa

## Abstract

**Background:**

The clinical, radiological and microbiological features of culture-confirmed childhood tuberculosis diagnosed at two referral hospitals are described.

**Methods:**

Cultures of *Mycobacterium tuberculosis *from children less than 13 years of age at Tygerberg and Red Cross Children's Hospitals, Cape Town, South Africa, were collected from March 2003 through February 2005. Folder review and chest radiography were performed and drug susceptibility tests done.

**Results:**

Of 596 children (median age 31 months), 330 (55.4%) were males. Of all children, 281 (47.1%) were HIV-uninfected, 133 (22.3%) HIV-infected and 182 (30.5%) not tested. Contact with infectious tuberculosis adults was recorded in 295 (49.5%) children. Missed opportunities for chemoprophylaxis were present in 117/182 (64.3%) children less than 5 years of age.

Extrathoracic TB was less common in HIV-infected than in HIV-uninfected children (49/133 vs. 156/281; odds ratio 0.50, 95% confidence interval 0.32–0.78). Alveolar opacification (84/126 vs. 128/274; OR 1.85, 95%CI 1.08–3.19) and cavitation (33/126 vs. 44/274; OR 2.28, 95%CI 1.44–3.63) were more common in HIV-infected than in HIV-uninfected children. Microscopy for acid-fast bacilli on gastric aspirates and sputum was positive in 29/142 (20.4%) and 40/125 (32.0%) children, respectively. Sixty-seven of 592 (11.3%) children's isolates showed resistance to isoniazid and/or rifampicin; 43 (7.3%) were isoniazid-monoresistant, 2 (0.3%) rifampicin-monoresistant and 22 (3.7%) multidrug-resistant. Death in 41 children (6.9%) was more common in HIV-infected children and very young infants.

**Conclusion:**

HIV infection and missed opportunities for chemoprophylaxis were common in children with culture-confirmed TB. With cavitating disease and sputum or gastric aspirates positive for acid-fast bacilli, children may be infectious. Transmission of drug-resistant TB is high in this setting.

## Background

In recent years there has been increasing recognition of the significant contribution of childhood tuberculosis (TB) to the global burden of TB [1,2]. This increased international awareness is evidenced by the recent publication of childhood TB guidance for National TB Programs published by the World Health Organization (WHO) and the access provided to child friendly drug formulations via the Global Drug Facility (GDF) in 2007 [3]. Children less than 15 years of age constitute approximately 15% of the total TB case load in many developing communities [4,5]. Children are mainly infected by adult pulmonary TB source cases and childhood TB therefore reflects the intensity of ongoing transmission of *Mycobacterium tuberculosis *(*M. tuberculosis*) within a community. The high prevalence of human immunodeficiency virus (HIV) infection in many TB-endemic countries fuels the TB epidemic and complicates the diagnosis of childhood TB.

Although the diagnosis of tuberculosis in the majority of children who present with symptomatic disease should not be that difficult, if a constellation of history of contact, chronic symptoms and special investigations such as tuberculin skin test (TST) and chest radiography are used [6], bacteriologic confirmation of the diagnosis is often lacking. Specimens for culture of *M. tuberculosis *in children, especially infants and young children are difficult to obtain and are therefore often restricted to hospital settings.

In Cape Town and the surrounding Western Cape Province, the majority of specimens positive on culture for *M. tuberculosis *in children are collected in two referral hospitals, Tygerberg Children's Hospital (TCH) and Red Cross Children's Hospital (RCCH). This study describes the clinical, radiological and microbiological features of all culture-confirmed childhood TB cases, comparing HIV-uninfected with HIV-infected children, diagnosed at these two hospitals over a 2 year period.

## Methods

### Setting

This prospective study was conducted in the Western Cape Province of South Africa, which reported a TB incidence of 931 and 1037 per 100 000 population in 2003 and 2005, respectively [7]. The HIV prevalence among women attending public antenatal care facilities was 13.1% (95% CI: 8.5–17.7%) and 15.7% (95% CI: 11.3–20.1%) in 2003 and 2005, respectively [8].

### Study population

All cultures of *M. tuberculosis *from children less than 13 years of age diagnosed at TCH and RCCH, were collected prospectively from 1 March 2003 through 28 February 2005. Clinical assessment of children for culture of *M. tuberculosis *was done by hospital clinicians in a routine fashion. A folder review and chest radiograph evaluation was performed on every child.

### Drug susceptibility testing

Laboratory procedures were as follows: Middlebrook 7H9 broth based (Mycobacteria Growth Indicator Tubes [MGIT]; Becton Dickinson, Sparks, MD, USA) culture medium was used for selective primary isolation of mycobacterial strains. *M. tuberculosis *was identified by polymerase chain reaction (PCR) DNA amplification method [9]. A single *M. tuberculosis *isolate from each patient was sent to the National Health Laboratory Service (NHLS) for drug susceptibility testing (DST). Initial screening was for isoniazid (INH) and rifampicin (RMP) resistance only; in case of resistance to either or both drugs, susceptibility testing for ethambutol was done. For TCH, drug susceptibility testing was by the indirect proportion method on Löwenstein-Jensen medium containing critical concentrations of 0.2 μg/ml INH, 30 μg/ml RMP and, where indicated, 2 μg/ml ethambutol as previously described [10]. DST for all RCCH isolates was initially performed using the Bactec 460TB system (Becton Dickinson, Sparks, MD, USA), according to international criteria [11]. Briefly, the susceptibility of a strain was judged by comparing growth of organisms in liquid media containing drug to growth in medium with no drug. RMP was tested at a concentration of 2.0 μg/ml, INH at 0.1 μg/ml and ethambutol at 7.5 μg/ml. Resistance was defined as 1% or more bacterial growth. DST by the indirect proportion method on Löwenstein-Jensen medium was repeated on all drug-resistant isolates and 20% of the drug-susceptible strains from RCCH. Quality assurance for drug susceptibility results was done locally with every batch and quarterly by the national tuberculosis reference laboratory as described previously [11,12].

### Clinical data and treatment

The clinical records of all children with a positive *M. tuberculosis *culture were reviewed. Data retrieved were demographic information, a history of close contact with an adult with smear or culture-positive pulmonary TB, a history of previous TB chemoprophylaxis or treatment, clinical features and weight at presentation, Mantoux (tuberculin skin test or TST) results, HIV status and whether antiretroviral therapy was started, TB disease manifestations recorded and hospital outcome.

Previous TB treatment was defined as more than 1 month of any antituberculosis treatment. Weight at admission was used to classify children as being well-nourished or having severe malnutrition (marasmus and/or kwashiorkor) according to the Wellcome classification [13]. The TST was considered positive with an induration of ≥ 10 mm in HIV-uninfected and ≥ 5 mm in HIV-infected children. HIV tests were requested at the discretion of the attending doctor. HIV infection was confirmed by two enzyme-linked immunosorbent assays (ELISA) in children ≥ 18 months old and by PCR in children younger than 18 months of age. All chest radiographs were read by a single paediatric TB specialist in a standardized manner [14].

Children with drug-susceptible TB were treated according to the directly observed treatment, short-course (DOTS) policy of the South African National Tuberculosis Programme (NTP), using INH, RMP and pyrazinamide (PZA) for 6 months [15]. Children with drug-resistant TB received individualized treatment according to their specific drug susceptibility pattern [16].

Categorical data were analyzed using the Chi-square test. The Fisher's exact test was applied where appropriate. Statistical analysis was done using Epi-Info version 6.04. The study was approved by the Ethics Review Board, Health Sciences Faculty, Stellenbosch University and permission for the study was obtained from both hospitals.

## Results

Of the 596 children (55.4% males) with culture-confirmed tuberculosis, 323 were from TCH and 277 from RCCH (4 children had a positive culture during the same TB episode at both hospitals). The median age was 31 months (range 16 days-156 months). Thirty three (5.5%) were infants less than 3 months of age, 156 (26.2%) were less than 1 year and 425 (71.3%) children were less than 5 years of age at diagnosis. Previous treatment and contact history, clinical data and special investigations are summarized in Table 1.

**Table 1:** History, clinical characteristics and drug susceptibility test results in children with culture-confirmed TB according to HIV status (n = 596)

Characteristic	HIV-uninfected N = 281 (%)	HIV-infected N = 133 (%)	HIV unknown N = 182 (%)	Odds ratio (95% CI)^a^
Previous TB treatment	26 (9.3)	40 (30.1)	16 (8.8)	0.24 (0.13–0.42)
Known TB source case	152 (54.1)	73 (54.9)	70 (38.5)	NS
Nutritional status	(n = 276)	(n = 128)	(n = 173)^b^	
Severely malnourished	53 (19.2)	26 (20.3)	20 (11.6)	NS
< 3^rd ^percentile WFA	78 (27.8)	57 (42.9)	44 (24.2)	0.49 (0.31–0.78)
Total malnourished	131 (46.6)	83 (62.4)	64 (35.2)	0.49 (0.31–0.77)
TST (Mantoux) positive	190/232 (81.9)	50/83^c ^(60.2)	90/110 (81.8)	2.99 (1.66–5.38)
DST results	(n = 277)^d^			
All drug resistance	31 (11.2)	23 (17.3)	13 (7.1)	NS
INH monoresistance	21 (7.5)	12 (9.0)	10 (5.5)	NS
RMP monoresistance	0	2 (1.5)	0	
MDR	10 (3.6)	9 (6.8)	3 (1.6)	NS
TB manifestation:				
Intrathoracic TB only^e^	126 (44.8)	84 (63.2)	96 (52.7)	0.47 (0.30–0.74)
Intra- and extrathoracic TB^e^	119 (42.3)	44 (33.1)	48 (26.4)	NS
Extrathoracic TB only	37 (13.2)	5 (3.8)	37 (20.3)	3.07 (1.41–11.48)
All extrathoracic TB	156 (55.5)	49 (36.8)	85 (46.7)	2.12 (1.36–3.32)

CI = confidence interval; WFA = weight for age; TST = tuberculin skin test; DST = drug susceptibility test; INH = isoniazid; RMP = rifampicin; MDR = multidrug-resistant

a: comparison between HIV-uninfected and HIV-infected groups

b: significantly fewer children were malnourished among those not HIV tested compared to those tested for HIV (OR 1.91; 1.30–2.80)

c: only 2 with TST 5–9 mm induration

d: in 4 HIV-uninfected children DST was not done

e: intrathoracic TB includes pleural and pericardial effusions

NS = not significant

### HIV status

HIV status was evaluated in 414 (69.5%) children. Of all children, 281 (47.1%) were confirmed HIV-uninfected, 133 (22.3%) were HIV-infected and in 182 (30.5%) the status was not known (in 2 cases mothers were known to be HIV-infected). Only 14 of the 133 (10.5%) HIV-infected children were on antiretroviral therapy at the time of TB diagnosis.

### History of TB contact and previous treatment

A history of contact with an infectious pulmonary TB case was recorded in 295 (49.5%) children. The source case was a parent in 121 (41.0%), an uncle/aunt in 68 (23.1%), a grandparent in 26 (8.8%), an older sibling in 15 (5.1%), other household cases in 27 (9.2%), a neighbour in 19 (6.4%) and another or unknown case in 28 (9.5%). Twenty three children (7.8%) had more than one source case. Two children, whose case histories have previously been published, were infected in a kangaroo care unit by a parent of another child [17] and one child was most likely infected by patients visiting their house for directly observed treatment (DOT), the mother being a DOT supporter. Missed opportunities for chemoprophylaxis, defined as no previous antituberculosis treatment and a known household source case in children less than 5 years of age, were present in 117 of 182 (64.3%) children.

Eighty two children (13.8%) had received previous TB treatment; 13 had received chemoprophylaxis, 11 were currently on treatment for > 1 month and 58 had received a previous course of treatment.

### Disease manifestations by HIV status

Extrathoracic TB was common in all groups. Table 2 reflects the extrathoracic TB manifestations recorded, according to the HIV status of the child. Significantly less extrathoracic TB was seen in HIV-infected children compared to HIV-uninfected (OR 0.50, 95% CI 0.32–0.78). Specific differences in subtype of extrathoracic TB observed more commonly in HIV-uninfected than HIV-infected children were in TB meningitis (68/281 vs. 10/133; OR 3.93, 95% CI 1.87–8.44) and osteoarticular TB (1/281 vs. 5/133; p = 0.014). Congenital TB occurred in 6 (1.0%) infants; 3 in HIV-uninfected, 1 HIV-infected and 2 with unknown HIV status.

**Table 2:** Extrathoracic disease manifestations based on HIV status in children with culture-confirmed tuberculosis

Extrathoracic disease manifestation	All children N = 596 (%)	HIV-uninfected N = 281 (%)	HIV-infected N = 133 (%)	HIV unknown N = 182 (%)
Miliary TB	69 (11.6)	44 (15.7)	19 ^a ^(14.3)	6 (3.3)
TB meningitis ^e^	89 (14.9)	68 (24.2)	10 (7.5)	11 (6.0)
Miliary TB & TB meningitis ^b^	25 (4.2)	18 (6.4)	6 (4.5)	1 (0.5)
Abdominal TB	43 (7.2)	24 (8.5)	14 (10.5)	5 (2.7)
Lymph node TB ^c^	94 (15.8)	40 (14.2)	13 (9.8)	41 (22.5)
Osteoarticular TB ^d, f^	18 (3.0)	1 (0.2)	5 (3.8)	12 (6.6)
Mastoid & ear TB	9 (1.5)	2 (0.7)	3 (2.3)	4 (2.2)
Mastoiditis	7	2	1	4
Chronic otorrhoea	2	0	2	0
Parotid gland	3 (0.5)	0	0	3 (1.6)
Renal TB	1 (0.2)	0	1 (0.8)	0
Skin involvement	8 (1.3)	3 (1.1)	0	5 (2.7)

a: Lymphoid interstitial pneumonitis excluded by treatment response

b: Included in miliary TB and TB meningitis above

c: All histologic or culture confirmed. A further 10 children had suspected TB lymph nodes

d: Orthopaedic patients: Spinal TB (6), hip TB (5), skull lesion (3), knee TB (2)

e: HIV-uninfected vs. HIV-infected group: odds ratio 3.93 (95% confidence interval 1.87–8.44)

f: HIV-uninfected vs. HIV-infected group: p = 0.014

Presenting symptoms were not always available in the records. Cough > 2 weeks was noted in 344 (57.7%) (337/517; 65.2% of those with intrathoracic disease), weight loss or failure to gain weight in 318 (53.4%) and fever in 284 (47.7%). Other symptoms noted were associated mainly with extrathoracic TB; neck mass (78; 13.1%), loss of consciousness (34; 5.7%), convulsions (27; 4.5%) and abdominal distension (26; 4.4%).

Table 3 demonstrates the intrathoracic TB manifestations seen on chest radiography (n = 555) according to the HIV status of the child. A significantly higher proportion of alveolar opacification (OR 1.85, 95%CI 1.08–3.19) and cavitation (OR 2.28, 95%CI 1.44–3.63) was recorded in HIV-infected compared to HIV-uninfected children and fewer HIV-infected children had normal chest radiographs (OR 0.31, 95%CI 0.10–0.87). There was no significant difference in the occurrence of cavitation by age group (0–2 years vs. > 2 years; 0–5 years vs. > 5 years or 0–9 years vs. > 9 years of age). Cavitation in younger children was mainly breakdown in a consolidated segment or lobe, while older children had more adult-type TB.

**Table 3:** Intrathoracic disease manifestations on chest radiograph based on HIV status in children with culture-confirmed tuberculosis

Intrathoracic disease manifestation^b^	All children^a ^n = 555 (%)	HIV-uninfected n = 274 (%)	HIV-infected n = 126 (%)	HIV unknown n = 155 (%)
Lymphadenopathy (all)	288 (51.9)	142 (51.8)	71 (56.3)	75 (48.4)
Paratracheal only	18 (3.2)	9	5	4
Perihilar only	192 (34.6)	91	46	55
Both	78 (14.1)	42	20	16
Large airway compression	97 (17.5)	54 (19.7)	17 (13.5)	26 (16.8)
Perihilar opacification	38 (6.8)	15 (5.5)	8 (6.3)	15 (9.7)
Alveolar opacification lobe/segment ^e^	285 (51.4)	128 (46.7)	84 (66.7)	73 (47.1)
Bronchopneumonic opacification	55 (9.9)	28 (10.2)	12 (9.5)	15 (9.7)
Collapse lobe/segment	52 (9.4)	23 (8.4)	14 (11.1)	15 (9.7)
All cavities/breakdown^f^	106 (19.1)	44 (16.1)	33 (26.2)	29 (18.7)
Pleural effusion (all)	83 (15.0)	37 (13.5)	17 (13.5)	29 (18.7)
Large effusions	29 (5.2)	9 (3.3)	5 (4.0)	15 (9.7)^c^
Pericardial effusion	11 (2.0)	6 (2.1)	3 (2.3)	2 (1.1)
Miliary picture	69 (12.4)	44 (16.1)	19 (15.1) ^d^	6 (3.9)
Ghon focus	26 (4.7)	18 (6.6)	3 (2.4)	5 (3.2)
Hyperinflation	15 (2.7)	6 (2.2)	5 (4.0)	4 (2.6)
Calcification	13 (2.3)	4 (1.5)	4 (3.2)	5 (3.2)
Normal chest radiograph^g^	53 (9.5)	32 (11.7)	5 (4.0)	16 (10.3)

a: 41 chest radiographs not done (extrapulmonary TB) or lost

b: In case of uncertainty about a characteristic, it is reported as not present

c: Older children had more large effusions and were less often HIV tested.

d: A further 8 patients had lymphoid interstitial pneumonitis

e: HIV-uninfected vs. HIV-infected group: odds ratio 0.44 (95% confidence interval 0.28–0.70)

f: HIV-uninfected vs. HIV-infected group: OR 0.54 (95% CI 0.31–0.93)

g: HIV-uninfected vs. HIV-infected group: OR 3.20 (95% CI 1.15–9.60)

### Culture source and microscopy results

Of 935 culture-positive isolates, 740 (79.1%) were pulmonary specimens (440 gastric aspirates, 202 sputum, 57 tracheal/bronchial aspirates and 41 nasopharyngeal aspirates). Lung or mediastinal lymph node biopsy yielded an additional 10 positive cultures. Of 185 (19.8%) culture-positive extrapulmonary specimens, 74 were from peripheral lymph nodes, 46 from cerebrospinal fluid, 13 from pleural and 4 from pericardial fluid, 15 from bone/sinovial tissue, 6 from ascites/abdominal tissue, 10 from mastoid or ear swabs, 3 from parotid aspirates, 1 from eye tissue and 13 from abscess pus swabs.

Staining and microscopy for acid-fast bacilli (AFB) on respiratory secretions (sputum, induced sputum, gastric aspirates and nasopharyngeal aspirates) was done in 309 children. AFB on gastric aspirates was positive in 29 of 142 (20.4%) children. AFB yield on self-expectorated sputum was 34 of 92 (37.0%) and 40 of 125 (32.0%) if induced sputum was included.

### Drug susceptibility test results

Drug susceptibility testing was done on isolates of 592 (99.3%) children. In 4 cases isolates were lost or contaminated. The results of the DST from TCH were previously published [18]. Of the 592 isolates, 67 (11.3%) showed resistance to INH and/or RMP; 43 (7.3%) were INH-monoresistant, 2 (0.3%) were RMP-monoresistant and 22 (3.7%) were MDR. Three INH-monoresistant isolates from RCCH by the Bactec DST method showed no resistance by the Löwenstein-Jensen DST method, while all retested drug-susceptible isolates showed the same result as with Bactec DST method. For consistency of results, the Löwenstein-Jensen DST results were accepted. The overall number of resistant cases did not differ significantly between the two hospitals (39/315, 12.4% from TCH vs. 28/277, 10.1% from RCCH; odds ratio 0.80, 95% confidence interval 0.46–1.37). There was no significant difference in the proportion of HIV-uninfected and infected cases with drug resistance.

Drug-resistance was significantly more in previously treated children (20/82; 24.4%) compared to children with no or unknown previous TB treatment (47/514; 9.1%. OR 0.31, 95% CI 0.17–0.59). DST results were known from only 18 source cases; 6 had MDR TB (5 children with MDR TB), 1 was RMP mono-resistant, as was the child, and 11 had drug-susceptible TB (10 children with drug-susceptible TB, 1 with MDR TB).

### Management and outcome

Treatment initiation was documented in 570 (95.6%) children; 380 (63.8%) were referred to their local health clinic for treatment, 98 (16.4%) were admitted to TB hospitals, 34 (5.7%) were transferred back to the hospital from where they were referred, 8 (1.3%) remained in the same hospital, in 9 (1.5%) cases the place of transfer was not noted and 41 (6.9%) children died. Of those admitted to TB hospitals for treatment, 81 (82.7%) had severe extrapulmonary TB; TB meningitis in 54, miliary TB in 10, abdominal TB in 18, pericardial effusion in 4 and spinal TB in 2 (some > 1 type). Thirteen of 98 (13.26%) admissions were drug-resistant cases (10 MDR) and 24 (24.5%) were HIV-infected. In total 41 died; 18/281 (6.4%) HIV-uninfected, 17/133 (12.8%) HIV-infected, and 6/182 (3.3%) in whom the HIV status was unknown. Significantly more HIV-infected children died compared to the other groups (OR 2.14, 95% CI 1.01–4.54 and 4.30, 1.54–12.61, respectively). Further, 9/67 (13.4%) (6 INH-monoresistant, 2 also HIV-infected, and 3 MDR, all 3 HIV-infected) children with drug-resistant TB died compared to 32/525 (6.1%) with known drug-susceptible TB (OR 2.39, 95% CI 1.00–5.55). Death was also significantly related to young age (Table 4) and 8 of 33 (24.2%) infants less than 3 months died compared to 33/563 (5.9%) children more than 3 months of age (OR 5.35, 95% CI 2.03–13.75). Of those less than 3 months of age that died, 4 had congenital TB, 3 had miliary and 1 bilateral bronchopneumonic TB, 2 were HIV-infected and 2 had INH-resistant TB. Death occurred within the first 2 weeks after admission in 18/41 (43.9%) and within 3 months following admission in 32/41 (78.0%).

**Table 4:** Clinical and laboratory features of children with culture-confirmed TB, compared in relevant age groups

Feature	Age < 3 years n = 334 (%)	Age > 3 years n = 262 (%)	Odds ratio (95% CI)
History of TB contact	183 (54.8)	112 (42.7)	1.62 (1.16–2.20)
Parent with TB	83 (24.9)	38 (14.5)	1.95 (1.25–3.05)
Previous TB treatment	33 (9.9)	49 (18.7)	0.48 (0.29–0.79)
Malnutrition/WFA < 3^rd ^centile	170/320 (53.1)	107/258 (41.5)	1.60 (1.13–2.26)
Tuberculin skin test ≥ 10 mm	176/238 (73.9)	151/187 (80.7)	NS
All pulmonary TB	303 (90.7)	214 (81.7)	2.19 (1.32–3.66)
All extrapulmonary TB	144 (43.1)	164 (62.6)	0.45 (0.32–0.64)
Miliary [also TB meningitis]	43 (12.9) [12 (3.6)]	26 (9.9) [13 (5.0)]	NS
TB meningitis	49 (14.7)	40 (15.3)	NS
Pleural effusion (large)	4 (1.2)	25 (9.5)	0.11 (0.03–0.35)
Lymph node TB	42 (12.6)	52 (19.8)	0.58 (0.36–0.93)
Abdominal TB	15 (4.5)	28 (10.7)	0.39 (0.20–0.78)
Osteoarticular TB	3 (0.9)	15 (5.7)	0.15 (0.03–0.56)
Other EPTB	10 (3.0)	23 (8.8)	0.32 (0.14–0.72)
HIV test done	258 (77.2)	156 (59.5)	2.31 (1.59–3.34)
HIV-infected (of those done)	77 (29.8)	56 (35.9)	NS
Outcome – Died	31 (9.3)	10 (3.8)	2.58 (1.19–5.74)
Chest radiographic features	n = 299	n = 256	0.20 (0.07–0.51)
Lymphadenopathy	175 (58.5)	113 (44.1)	1.79 (1.26–2.54)
Airway compression	86 (28.8)	11 (4.3)	8.99 (4.52–18.33)
Alveolar opacification	179 (59.9)	106 (41.4)	2.11 (1.48–3.01)
Expansile pneumonia	25 (8.4)	9 (3.5)	2.50 (1.09–5.90)
Bronchopneumonic opac	33 (11.0)	22 (8.6)	NS
Collapse lobe/segment	31 (10.4)	21 (8.2)	NS
Cavities	55 (18.4)	51 (19.9)	NS
Ghon focus	17 (5.7)	9 (3.5)	NS
Hyperinflation	17 (5.7)	0	P < 0.001
Calcification	1 (0.3)	12 (4.7)	P = 0.002
Normal chest radiograph	26 (8.7)	28 (10.9)	NS

WFA = weight for age; NS = not significant

## Discussion

To our knowledge this is the largest cohort of culture-confirmed childhood TB described in the literature. It must be emphasized that this hospital-based study probably represents a biased population of sicker children, but we believe it should provide a good overview of children with definite (culture confirmed) TB who present to referral hospitals in TB-endemic settings.

Young and/or HIV-infected children carried the brunt of the TB disease burden; the age distribution (> 70% of children less than 5 years of age) reflects a high rate of recent *M. tuberculosis *transmission in this setting. Current NTP and WHO guidelines for the management of children in close contact with an infectious adult source case recommend chemoprophylaxis for children less than 5 years of age [3,15]. In this study, missed opportunities for chemoprophylaxis were present in the majority, and represented a key opportunity for intensified TB control in children. A source case was identified more often in young children (< 3 years of age) and a parent was the source case in many of these children.

From the few children in whom the DST results of the source cases were known, it is clear that the DST results of a probable adult source case are highly relevant and should guide the initial management of child contacts [3,16]. However, due to resource constraints culture and DST are rarely performed in TB-endemic areas, which frequently delays initiation of the correct treatment in childhood contacts or prevents even their identification.

Current (2006) WHO childhood TB guidelines recommend that all children suspected of having TB in a high HIV prevalence area or at high risk of HIV infection should be tested for HIV, as this may guide clinical management [3]. Although about 70% of children in the study were tested for HIV, many children in this high HIV prevalence area were not tested, even in some cases where their mothers were known to be HIV-infected. Knowing the HIV status is important, because several other HIV related conditions may mimic TB in HIV-infected children, which makes the diagnosis of TB more complex. HIV infection was confirmed in 32.1% of those tested, indicating the high risk of TB in HIV-infected children. This probably represents an overestimation of HIV prevalence among childhood TB patients in general, due to the patient selection alluded to earlier and the fact that children with symptoms or signs suspicious of HIV infection would have been more likely to have been tested. Even though the recorded HIV prevalence is very high, it is far lower than that recorded in adult TB patients from the same area. It illustrates, however, the need for integrated TB/HIV diagnosis and care in children.

Despite TST results being significantly more often positive in HIV-uninfected children compared to HIV-infected children (Table 1), the latter group had a remarkably high proportion (60.2%) of positive TST results, which confirms its value as a diagnostic tool even in HIV-infected children.

Lung cavities were not uncommon (19.1%) and were significantly more common in HIV-infected than HIV-uninfected children, which confirms findings in earlier studies in children [19,20]. Other underlying lung conditions such as bronchiectasis or previous staphylococcal infections could partly be responsible for cavities being more common in HIV-infected children. Cavities are associated with a high mycobacterial load and therefore these children pose a transmission risk [21].

Miliary TB was more common in HIV-uninfected compared to HIV-infected children, which differs from previous reports [19,20]. Extrathoracic TB, especially TB meningitis, was less common in HIV-infected children compared to HIV-uninfected children, which could reflect a reduction in immune mediated pathology in these children or perhaps that HIV infection precedes childhood TB infection (unlike adults, where TB infection often precedes HIV infection and dissemination of organisms may already have occurred by the time they are infected with HIV) and the rapidity of disease onset does not give time for dissemination and development of disease at sites other than the lungs. The finding is consistent with previous reports and also with the observation that HIV-infected children are less likely to develop the pathognomonic basal enhancement seen in immune competent children with TB meningitis [22,23].

This is also the largest cohort of children in whom DST has been done and is an accurate reflection of current transmission patterns. Drug resistance was common with 11.3% resistance to INH and/or RMP. This is significantly higher than a previous survey done at TCH from 1994–1998 (all resistance 21/306; 6.9%. OR 0.58, 95% CI 0.34–0.99) [10]. Despite the significantly higher number of HIV-infected children who had received previous TB treatment, there was no significant increase in drug resistance in HIV-infected children compared to HIV-uninfected children. However, previously treated children had significantly more drug-resistant TB than children with new TB, but as shown in our previous report on drug surveillance from TCH during the same period, the majority of these children had transmitted drug resistance, which was initially missed [18].

The study confirms the wide spectrum of TB disease manifestations seen in children who present to a referral hospital. TB was cultured from nearly every organ; however renal disease was the rarest manifestation. TB meningitis and miliary TB was common before and after 3 years of age, which could reflect referral bias or the fact that specimens for culture are more easily obtained in older children. Large TB pleural effusions were rarely seen before age 3 years and other forms of extrathoracic TB were also significantly more common in children > 3 years of age (Table 4). This is consistent with the literature. Several chest radiographic features were significantly more common in children < 3 years of age (Table 4), especially nodal airway compression. Fifty-one of 97 (52.6%) children with airway compression were less than 12 months of age.

Death as a hospital outcome measure was significantly more common in HIV-infected children and those with drug-resistant TB, although 5 of 9 drug-resistant cases who died were also HIV-infected. Only a minority of HIV-infected children received antiretroviral therapy at the time. Improved access to antiretroviral therapy should decrease the incidence of disease and improve the outcome of TB in HIV-infected children.

## Conclusion

Missed opportunities for chemoprophylaxis in children less than 5 years of age were common in this study group. The occurrence of HIV infection was high (32.1% of those tested) in children with culture-confirmed TB. Extrathoracic TB was less common in HIV-infected children with confirmed TB compared to HIV-uninfected children. With cavitating disease and sputum or gastric aspirates positive for acid-fast bacilli, children may be infectious. The prevalence of INH and/or RMP resistance in the study reflects a high rate of transmission of drug-resistant TB in this setting.

## Competing interests

The author(s) declare that they have no competing interests.

## Authors' contributions

All the authors 1) have made substantial contributions to conception and design, or acquisition of data, or analysis and interpretation of data; 2) have been involved in drafting the manuscript or revising it critically for important intellectual content; and 3) have given final approval of the version to be published.

## Pre-publication history

The pre-publication history for this paper can be accessed here:


